# A Case Report of High 18F-FDG PET/CT Uptake in Progressive Transformation of the Germinal Centers

**DOI:** 10.1097/MD.0000000000000412

**Published:** 2015-06-12

**Authors:** Chian A. Chang, Beena Kumar, Deepali Nandurkar

**Affiliations:** From the Department of Nuclear Medicine (CAC, DN); and Department of Anatomical Pathology (BK), Monash Health, Melbourne, Australia.

## Abstract

Progressive transformation of the germinal centers (PTGC) is a benign reaction pattern in lymph nodes. An association with Hodgkin disease (HD) has been reported and PTGC may precede, coexist, or present after the diagnosis of HD.

This case report describes a patient who presented with unprovoked pulmonary embolism and subsequent investigations showed a solitary abdominal mass, which was subsequently proven to be PTGC. PTGC is usually avid on fluorine-18-labeled fluorodeoxyglucose positron emission tomography with computed tomography for attenuation correction and may be mistaken for neoplastic disease. Being a reactive etiology, the metabolic activity is generally low with a low maximum standardized uptake value (SUVmax), but in our case, the metabolic activity and corresponding SUVmax were relatively high making the diagnosis difficult, as most clinicians would consider a high metabolically active process more likely malignant.

Recognition of PTGC is important, as it is not a malignant process. Owing to its reported associations, however, patients with this diagnosis will likely require regular surveillance.

## INTRODUCTION

Progressive transformation of the germinal centers (PTGC) is a benign reaction pattern in lymph nodes. The entity is characterized histologically by large reactive follicles with the mantle zone small B-cells infiltrating into the germinal centers resulting in blurring of the mantle zone-germinal center interface. PTGC may be associated with Hodgkin disease (HD), most commonly the lymphocyte predominant subtype,^1–3^ but association with the nodular sclerosing and mixed types has also been reported.^4^ PTGC may precede, coexist, or present after the diagnosis of HD. As PTGC is a reactive process, the fluorine-18-labeled fluorodeoxyglucose positron emission tomography with computed tomography for attenuation correction (F-18 FDG PET/CT) uptake is usually of low-to-moderate grade. We report a case of PTGC, which presented as a diagnostic dilemma wherein the F-18 FDG PET/CT uptake was unusually high with review of the literature.

## CASE REPORT

A previously well 67-year-old male was presented to the emergency department with acute shortness of breath and chest discomfort. He had no significant medical or surgical history, was not on medications, and did not have a smoking history, recent travel history, or prolonged period of immobilization. There was no family history of malignancy.

**FIGURE 1:**
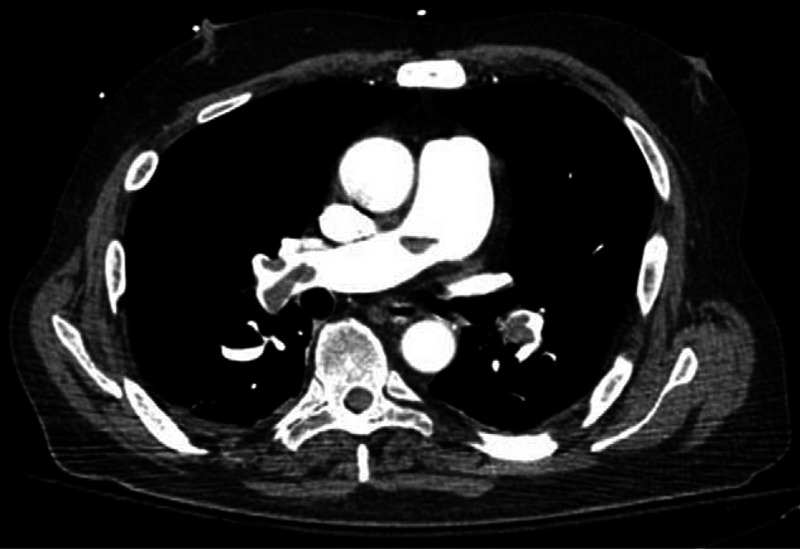
Computed tomography pulmonary angiogram shows multifocal filling defects in the pulmonary arterial system in keeping with acute bilateral pulmonary embolism.

On examination, he was mildly tachycardic at 110 beats per minute but regular, with a blood pressure of 130/80 mm Hg. He was tachypnoeic at 16 breaths per minute but afebrile. The remainder of his clinical examination was normal. A pulmonary embolus was suspected clinically and a computed tomography (CT) pulmonary angiogram was performed demonstrating multifocal filling defects in the pulmonary arterial system in both lungs mostly in the lower lobes confirming the diagnosis of acute bilateral pulmonary embolism (Figure 1). No complicating pulmonary infarction was seen on CT. He was admitted to the hospital and treated initially with subcutaneous enoxaparin injections and was commenced on warfarin.

**FIGURE 2:**
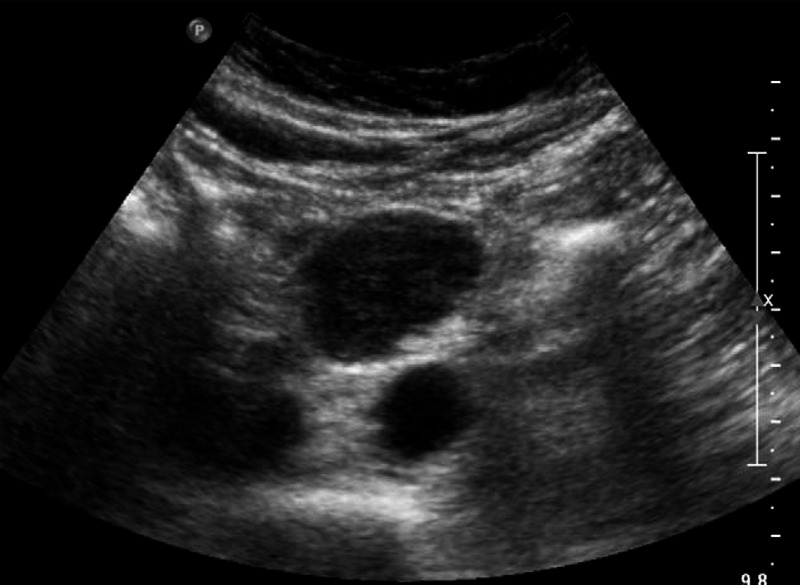
Abdominal ultrasound scan demonstrates an ovoid homogenous hypoechoeic mass anterior to the inferior vena cava and aorta in the mid abdomen.

**FIGURE 3:**
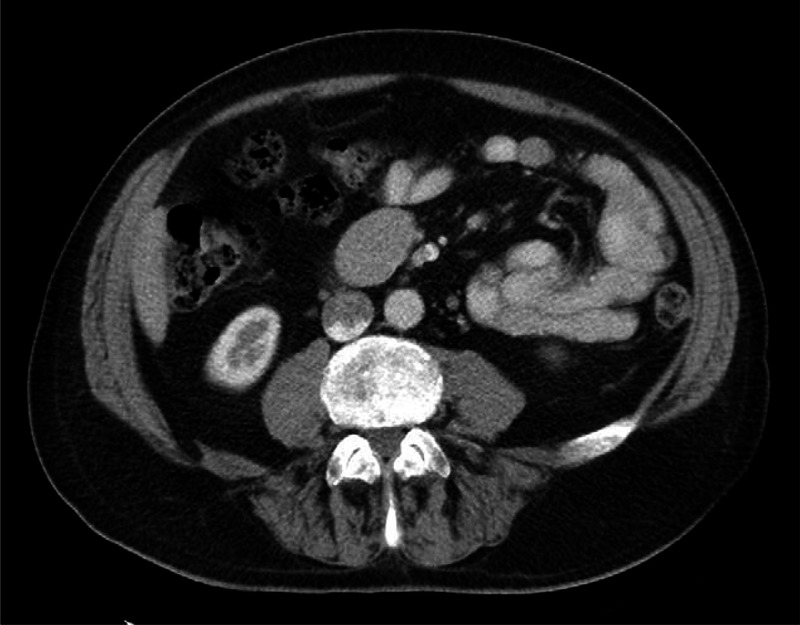
Abdominal computed tomography after administration of oral and intravenous iodinated contrast shows a homogenous enhancing mass in the root of the mesentry. No internal calcification or necrosis.

During his hospital admission, he was investigated for a cause of pulmonary embolism and serologic screening tests for coagulopathy including anti-thrombin III, Protein C, and Protein S deficiency were negative. A bilateral lower limb duplex ultrasound scan was then requested. Although no deep vein thrombosis was identified in the lower limb and pelvic veins including the lower inferior vena cava, a mass measuring 4.9 × 3.0 cm was identified in the root of the mesentery (Figure 2). Subsequent CT of the abdomen after administration of oral and intravenous contrast (Figure 3) confirmed a solitary soft tissue attenuating mass in the mid abdomen. No central necrosis or calcification was identified.

**FIGURE 4:**
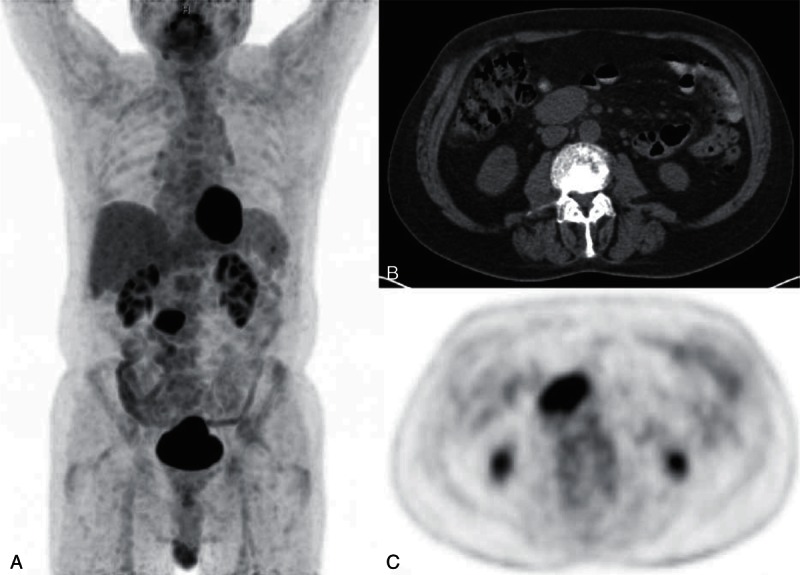
F-18 FDG PET/CT maximum intensity projection (A), CT for attenuation correction (B), and PET (C) images show the mass was intensely FDG avid (SUVmax 6.6). No enlarged or FDG avid lymph node was present elsewhere above or below the diaphragm. F-18 FDG PET/CT = fluorine-18-labeled fluorodeoxyglucose positron emission tomography with computed tomography for attenuation correction.

**FIGURE 5:**
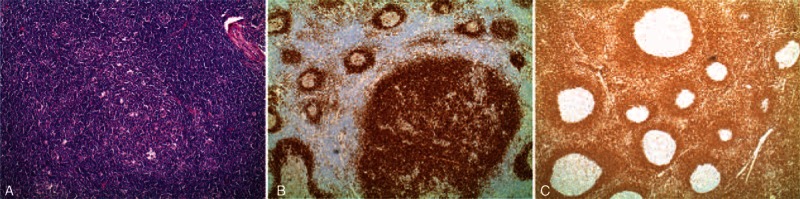
Histopathology examination shows an increased number of variably sized and significantly enlarged follicles (A). There was infiltration and accumulation of the mantle zone lymphocytes within the larger follicles on the CD79a stain (B). There was BCL2 negativity within the follicle (C). L&H cells and classical Reed-Sternberg-like cells were absent.

The patient remained hemodynamically stable throughout his admission and he declined further investigations and procedures. As the international normalized ratio was therapeutic (2–3) at that stage, he was discharged home and was organized to have an F-18 FDG PET/CT and follow-up in the outpatient clinic. The F-18 FDG PET/CT a month later confirmed the mass was solitary and was intensely avid with a maximum standardized uptake value (SUVmax) of 6.6 (Figure 4). The patient again declined intervention or biopsy at that stage, as he remained well clinically. A follow-up F-18 FDG PET/CT was scheduled 6 months later, which showed a mild increase in metabolic activity of the mass (SUVmax 8.5,) but no change in size anatomically. The patient remained clinically asymptomatic, but given the mild increase in metabolic activity, there was increasing concern of a malignancy and the patient subsequently agreed to a laparoscopic excision biopsy. The procedure was uneventful and the mass was completely excised with clear and negative margins. No lymphadenopathy elsewhere was identified intraoperatively. Histologic analysis confirmed the mass corresponded to a pathologically enlarged lymph node. It showed an increased number of variably sized, significantly enlarged follicles throughout the lymph node with the germinal centers in larger follicles showing irregular blurred margins. There was obvious infiltration and accumulation of the mantle zone lymphocytes within the larger follicles on the CD79a stain with BCL2 negativity, thus excluding follicular lymphoma. Flow cytometry studies showed no evidence of a monoclonal population or evidence of light chain restriction (Figure 5). L&H cells and classical Reed-Sternberg-like cells were absent. Based on these findings, a diagnosis of PTGC was made.

## DISCUSSION

PTGC is a benign process characterized by large reactive follicles with the mantle zone small B-cells infiltrating into the germinal centers resulting in a blurred mantle zone-germinal center interface. It may occur in up to 15% of enlarged lymph nodes with reactive follicular hyperplasia.^1^ It was first described in 1975 by Lennert et al^5^ and is considered a reactive condition, but can grow, spread, or even recur after resection.^6^ There is an association with HD, most commonly the lymphocyte predominant subtype,^1–3^ but association with the nodular sclerosing and mixed types has also been reported.^4^ PTGC may precede, coexist, or present after the diagnosis of HD. Shaik et al also found an association with immune disorders, including systemic lupus erythromatosis, Castleman disease, and autoimmune lymphoproliferative syndromes. Typically, a single lymph node is affected, although multiple lymph nodal involvement has also been reported. The commonest site to be involved is in the cervical region and less commonly, the axilla and inguinal regions. A male predominance has been observed and the incidence is higher in the adult population, although the chances of PTGC recurrence are higher in children.^1^

Several authors have reported cases of PTGC showing increased F-18 FDG PET/CT uptake, as it is a metabolically active process.^7,8^ Because of this, it may be mistaken for a neoplastic disease or recurrent disease when there is a history of treated lymphoma. A possible cause for PTGC positivity on F-18 FDG PET/CT is the strong expression of membrane-bound glucose transporter 1 (GLUT-1). This process of strong GLUT-1 expression, however, can be present in other cancer cells including HD.^9^ The metabolic activity of PTGC appears to be variable, ranging from 3.8 to 11.0,^8,10^ making the distinction between this entity and malignancy impossible. In our case, the SUVmax was relatively intense at 8.5 and most clinicians would associate high glycolytic activity with malignancy.

Recognition of this entity is important, as PTGC is a not a malignant process and is also not regarded premalignant. It may be mistaken for disease recurrence particularly in patients with a history of treated HD. In patients with no prior history, there is a small but potential risk of developing HD in the future. There are no anatomical or molecular imaging techniques capable of distinguishing PTGC from neoplastic disease; therefore, histologic analysis would be required to establish the diagnosis as was in this case. Presently, there are no consensus on the management of PTGC. Unless a systemic immunologic disease process is identified as a potential etiology, follow-up potentially with F-18 FDG PET/CT would be required to stage, monitor, and assess response to therapy, given the potential risk of developing HD in the future.
